# Improved nutritional status is related to improved quality of life in Parkinson’s disease

**DOI:** 10.1186/s12883-014-0212-1

**Published:** 2014-11-18

**Authors:** Jamie M Sheard, Susan Ash, George D Mellick, Peter A Silburn, Graham K Kerr

**Affiliations:** School of Exercise and Nutrition Sciences, Queensland University of Technology, Brisbane, Queensland Australia; Movement Neuroscience Program, Institute of Health and Biomedical Innovation, Queensland University of Technology, Brisbane, Queensland Australia; Australia Eskitis Institute for Cell and Molecular Therapies, Griffith University, Brisbane, Queensland Australia; University of Queensland Centre for Clinical Research, Brisbane, Queensland Australia

**Keywords:** Malnutrition, Parkinson disease, Quality of life, Nutritional status, Nutrition therapy

## Abstract

**Background:**

Quality of life is poorer in Parkinson’s disease than in other conditions and in the general population without Parkinson’s disease. Malnutrition also results in poorer quality of life. This study aimed at determining the relationship between quality of life and nutritional status.

**Methods:**

Community-dwelling people with Parkinson’s disease >18 years old were recruited. The Patient-Generated Subjective Global Assessment (PG-SGA) assessed nutritional status. The Parkinson’s Disease Questionnaire 39 (PDQ-39) measured quality of life. Phase I was cross-sectional. The malnourished in Phase I were eligible for a nutrition intervention phase, randomised into 2 groups: standard care (SC) with provision of nutrition education materials only and intervention (INT) with individualised dietetic advice and regular weekly follow-up. Data were collected at baseline, 6 weeks, and 12 weeks.

**Results:**

Phase I consisted of 120 people who completed the PDQ-39. Phase II consisted of 9 in the SC group and 10 in the INT group. In Phase I, quality of life was poorer in the malnourished, particularly for mobility and activities of daily living domains. There was a significant correlation between PG-SGA and PDQ-39 scores (Phase I, *r*_*s*_ = 0.445, *p =* .000; Phase II, *r*_*s*_ = .426, *p* = .002). In Phase II, no significant difference in the PDQ-39 total or sub-scores was observed between the INT and SC groups; however, there was significant improvement in the emotional well-being domain for the entire group, *X*^*2*^(2) = 8.84, *p* = .012.

**Conclusions:**

Malnourished people with Parkinson’s disease had poorer quality of life than the well-nourished, and improvements in nutritional status resulted in quality of life improvements. Attention to nutritional status is an important component of quality of life and therefore the total care of people with Parkinson’s disease.

**Trial registration:**

ACTRN12610000819022

## Background

Quality of life is positively associated with quality of health [1] and is therefore recognised as an important outcome for the treatment of many conditions, including Parkinson’s disease (PD). Reports about quality of life in PD indicate that it is poor compared to that of the general population [2,3] and in other conditions, [4,5] with virtually all aspects of the disease contributing to poorer quality of life. These include longer disease duration, [6] older age at disease onset, [7] more severe motor symptoms, [4,6,8,9] decreases in mobility [10] and the ability to carry out activities of daily living, [9,11,12] increases in the amount of medication required, [4,12] medication ineffectiveness, [4,13,14] dyskinesias, [13,15,16] pain, [8,10] problems with sleep, [4,8,10] cognitive impairment, [17] depression, [8-12,14] and anxiety [10,12,14].

Poor nutritional status, specifically malnutrition, results in a lower quality of life in the elderly [18,19] and other disease states [20-22]. People with PD are at risk of malnutrition; [23-25] therefore, it is possible that malnutrition in PD contributes to poorer quality of life, as a recent study demonstrated, with significant correlations between nutritional status and quality of life [26]. Factors affecting a person’s food intake, such as difficulties swallowing and loss of appetite, play an important role in quality of life [27]. Nutrition interventions aimed at improving these symptoms and, therefore, nutritional status can improve quality of life, as has previously been demonstrated in chronic kidney disease [22] and oncology [21,28,29] patients. Despite the extensive research related to quality of life in PD, there is limited evidence regarding the relationship between malnutrition and quality of life. There is also a paucity of information available about the results of nutrition interventions for malnutrition in PD in general and also as they affect quality of life.

The aim of the current study was to determine the relationship between nutritional status and quality of life in people with PD.

## Methods

The research was carried out in two phases. Informed written consent for both phases was obtained as per protocol approved by the Queensland University of Technology Human Research Ethics Committee.

In Phase I, community-dwelling people with idiopathic PD, aged >18 years, were recruited using a variety of methods between February and August 2011 [25]. Participants were excluded if they resided in an assisted living facility. Geographical location was limited to within ~2-hour driving distance of Brisbane, Queensland, Australia. PD diagnosis was determined by the participants’ physician or neurologist and was self-reported by the participant. Only a portion of the total data collected, as it relates to nutritional status and quality of life, is presented here, with other information regarding clinical status, motor symptoms, and non-motor symptoms reported previously [25,30].

For the majority of the participants, the assessments were conducted in their home, and the visit was scheduled to be within an hour of a PD medication dose or at a time when the participants stated that their function was best, to try to capture their function in an “On” state. The scored Patient-Generated Subjective Global Assessment (PG-SGA) was performed by a dietitian to assess nutritional status [31]. Nutrition assessment using the PG-SGA is based on a medical history (recent changes in weight, dietary intake, gastrointestinal symptoms, functional capacity, and disease status) and a physical examination of fat stores, muscle status, and fluid status. The assessment results in a categorisation of nutrition status: SGA-A (well nourished), SGA-B (moderately malnourished), or SGA-C (severely malnourished) [32], as well as a total score with a higher score indicating poorer nutritional status. The PG-SGA score places people into triage categories indicating the need for nutrition or medical intervention: 0-1 points (category 1, no intervention required), 2-3 points (category 2, patient and family education required), 4-8 points (category 3, requires intervention by dietitian), ≥9 (category 4, critical need for symptom management and/or nutrition intervention).

The Parkinson’s Disease Questionnaire (PDQ-39) [33] was completed to assess quality of life related to PD. A higher score indicates poorer quality of life. It was completed by the participants while the researcher was present in order that instructions could be provided that an answer of “Always” or “Cannot do at all” was appropriate if they no longer performed a task due to the difficulties associated with their PD. In the case of poor cognitive status, as assessed by the Addenbrooke Cognitive Examination, the spouse or caregiver was asked to assist with completing the questionnaire. Each of the PDQ-39 sub-scores was also calculated: mobility, activities of daily living (ADL), emotional well-being (EWB), stigma, social support, cognition, communication, and bodily discomfort. The Unified Parkinson’s Disease Rating Scale (UPDRS) motor sub-scale (III) was completed as previously described [30]. Birth date and self-reported disease duration (PD duration) were collected.

During Phase II, participants were considered eligible when they were assessed as being at risk of malnutrition or malnourished in Phase I using one of the following criteria: SGA category B or C, Mini-Nutritional Assessment (MNA) score ≤23.5, PG-SGA score ≥9, BMI <22 kg/m^2^ for participants aged ≥65 years, or BMI <18 kg/m^2^ for participants aged ≤64 years. The inclusion criteria included both those at risk of malnutrition and malnourished in order to follow the recommendation that those at risk of malnutrition should receive some intervention [34] and to provide a sample size of 20 participants, given the time limitations of the study. Exclusion criteria included current management by a dietitian, hospitalisation at the time of intervention, deep brain stimulation scheduled within the next 3 months, significant recent improvement in nutritional status prior to Phase I, intentional weight loss resulting in reduced intake and low MNA score, and MNA score ≤23.5 or BMI < age-specific cut-offs but no significant changes in diet history, anthropometry, recent weight history, or functional status and were deemed to be of normal nutritional status by the dietitian [35].

The participants were randomised to 2 groups (SC: standard care; INT: intervention) using a random number generator to assign participant numbers, and the group assignments were placed in sealed envelopes, with the number on the outside, by a research associate not associated with the project. Rolling recruitment was conducted to allow for Phase II to run concurrently with the end of Phase I. As eligible participants agreed to participate, they were assigned the next consecutive number, which identified the envelope to open to determine the group assignment.

The aim of Phase II was to improve nutritional status over a 12-week period. The SC group received written information only while the INT group received individualised nutrition information by a dietitian and weekly phone contact. The participants were informed of the groups, including that one would include more information and contact than the other. Each participant was involved in 4 visits: 1. Instruction visit (pre-BL) one week prior to the baseline time point; 2. Baseline measurements (BL); 3. 6-week measurements (6 wk); 4. 12-week measurements (12 wk). These visits were conducted at the same time every visit, similar to the Phase I timing for as close to an “On” state as possible, and the assessments were performed in the same order to minimise the effects of medication on the outcomes. Written consent was obtained at the pre-BL visit. The PDQ-39, SGA, and PG-SGA were completed at the BL, 6 wk, and 12 wk visits but not the pre-BL visit. The Hoehn and Yahr (H&Y) scale, which is a five-point scale (1-5) with a higher rating on the scale indicating a greater amount of disability and impairment, was recorded. The H&Y scores were dichotomized (less severe PD, H&Y 0-3; severe PD, H&Y 4-5). Current medications were collected at each of the 4 visits, and the levodopa equivalent doses (LEDD) were calculated per kg body weight (mg/kg). Changes in medication were at the treating physician’s or neurologist’s discretion and were not controlled in the study. All participants who required further intervention received referrals to a dietitian after completion of the project.

### Statistical analysis

Variables of interest were not normally distributed for the SGA-A group or for each of the PG-SGA triage categories. Therefore, median (range) values are reported when comparing by groups, and non-parametric Mann-Whitney U tests were conducted to compare the scores between PG-SGA categories. Non-parametric independent samples Kruskal Wallis tests were conducted to compare the scores between the PG-SGA triage categories. In Phase II, the Friedman’s Two-Way ANOVA by ranks was conducted to determine differences in PDQ-39 score for each group (SC, INT) as well as the entire sample over the intervention period. The comparison with the entire sample was conducted owing to the small sample size and the potential that participants improved their nutritional status as a result of knowledge of their nutritional status at baseline. Spearman’s correlations were conducted between PG-SGA and PDQ-39 scores and between PG-SGA score and each PDQ-39 sub-score. Median change in PDQ-39 score between BL and wk 12 was calculated for each group. These changes were compared between groups (SC, INT) using non-parametric Mann-Whitney *U* tests. A change of 2 points in the total score was considered clinically meaningful [36].

A maximum likelihood linear mixed model with random intercepts was used to analyse the longitudinal data with PDQ-39 as the outcome variable and PD duration, gender, dichotomized H&Y grouping, log-transformed PG-SGA scores, intervention group (SC, INT), time (BL, 6 wk, 12 wk), and group*time interaction as fixed effects.

Statistical analysis was completed using SPSS Version 19 (SPSS Inc., Chicago, IL, USA). Statistical significance was set at *p* <0.05.

## Results

### Phase I

One hundred twenty five community dwelling adults aged >18 years (74 M, 51 F) participated in Phase I. Median age of the participants was 70 (35–92) years. Self-reported median length of disease was 6 (0–31) years. The median H&Y score was 2 with 92.8% of participants in the less severe category and 7.2% in the more severe category. Nineteen (15.2%) participants were moderately malnourished (SGA-B), while none were severely malnourished (SGA-C). Further details about the sample are available elsewhere [25]. Briefly, the median UPDRS III score was 15 (1–37) in the SGA-A group and 20 (11–39) in the SGA-B group (*U* = 1376.5, *z* = 2.63, *p* = 0.008).

PDQ-39 scores were missing for 5 participants because those participants did not wish to complete the entire data collection visit. Therefore, analysis was only completed for 120 participants (103 SGA A, 17 SGA B) with a median age of 71 (35–92) years and median self-reported length of disease of 6 (0–26) years. Median PG-SGA score was 2 (0–15) for SGA-A and 8 (4–15) for SGA-B participants (*U* = 1611.5, *z* = 5.60, *p* = 0.000).

SGA-B participants had higher median PDQ-39 total and sub-scores than SGA-A participants. These differences were statistically significant between the groups in the mobility (*U* = 1195.00, *z* = 2.41, *p* = 0.016) and activities of daily living (*U* = 1128.5, *z* = 2.67, *p* = 0.008) sub-scores (Table 1).

**Table 1:** **Differences in PDQ-39 score and sub-scores between SGA categories**

	**SGA-A**	**SGA-B**	**PG-SGA score**
	**n = 103**	**n = 17**	**r**_**s**_ **, p value**
PDQ-39 total score	23 (3–66)	28 (10–50)	0.445, .000*
Mobility sub-score	28 (0–100)*	45 (2–85)*	0.520, .000*
Activities of Daily Living sub-score	21 (0–100)*	42 (8–79)*	0.412, .000*
Emotional Wellbeing sub-score	17 (0–92)	29 (0–58)	0.243, .008*
Stigma sub-score	12 (0–69)	12 (0–75)	0.150, .101
Social Support sub-score	0 (0–75)	8 (0–50)	0.317, .000*
Cognition sub-score	31 (0–69)	31 (6–56)	0.239, .009*
Communication sub-score	17 (0–75)	25 (8–58)	0.212, .020*
Bodily discomfort sub-score	25 (0–100)	33 (0–75)	0.186, .042*

*Abbreviations:* *PDQ-39* Parkinson’s Disease Questionnaire-39, *PG-SGA* Patient-Generated Subjective Global Assessment, *SGA* Subjective Global Assessment.

Values are reported as median (range) and compared using Mann-Whitney *U* tests and correlations between total PG-SGA score with PDQ-39 score and sub-scores measured by Spearman’s correlation in Phase I.

*Statistically significant differences between groups, *p* < .05.

PDQ-39 score was significantly different between triage categories (*X*^*2*^(3) = 22.03, *p* = 0.000) (Table 2) with post-hoc tests indicating that the differences were between triage categories 1 and 3 (*z* = -3.94, *p* = 0.000) and triage categories 1 and 4 (*z* = -3.65, *p* = 0.002). Significant differences also existed in the mobility sub-score (*X*^*2*^(3) = 36.06, *p* = 0.000). Post-hoc analysis revealed differences between categories 1 and 3 (*z* = -4.17, *p* = 0.000), categories 1 and 4 (*z* = -5.50, *p* = 0.000), and categories 2 and 3 (*z* = -3.21, *p* = 0.008). The ADL sub-score was also significantly different (*X*^*2*^(3) = 22.13, *p* = 0.000). Post-hoc analysis indicated that these differences existed between categories 1 and 3 (*z* = -3.89, *p* = 0.001) and categories 1 and 4 (*z* = -3.295, *p* = 0.001). Finally, the social support sub-score was also significantly different between the categories (*X*^*2*^(3) = 10.25, *p* = 0.017). Post-hoc analysis resulted in differences between categories 1 and 3 only (*z* = -2.72, *p* = 0.040).

**Table 2:** **Differences in PDQ-39 score and sub-scores between PG-SGA triage categories**

	**PG-SGA triage categories**			
	**Triage category 1**	**Triage category 2**	**Triage category 3**	**Triage category 4**
	**(n = 35)**	**(n = 37)**	**(n = 32)**	**(n = 16)**
PDQ-39 total score	17*	20	30.5*	28*
	(3 - 52)	(5 - 58)	(10 - 66)	(16 - 64)
Mobility	5*	28*	45*	43.5*
	(0 - 68)	(0 - 79)	(2 - 100)	(10 - 85)
Activities of daily living	17*	21	31*	40*
	(0 - 50)	(0 - 79)	(4 - 100)	(12 - 83)
Emotional wellbeing	8	17	31	23
	(0 - 58)	(0 - 54)	(0 - 92)	(0 - 75)
Stigma	12	12	12	19
	(0 - 56)	(0 - 62)	(0 - 75)	(0 - 69)
Social support	0*	0	8*	10
	(0 - 50)	(0 - 50)	(0 - 75)	(0 - 75)
Cognition	25	31	31	41
	(0 - 69)	(0 - 69)	(0 - 62)	(6 - 62)
Communication	17	17	25	25
	(0 - 67)	(0 - 67)	(0 - 75)	(0 - 67)
Bodily discomfort	25	33	29	42
	(0 - 75)	(0 - 75)	(0 - 75)	(0 - 100)

*Abbreviations:* *PDQ-39* Parkinson’s Disease Questionnaire-39, *PG-SGA* Patient Generated Subjective Global Assessment.

Values reported as median (range) and compared using Kruskal Wallis tests.

Triage category 1: No intervention required; Triage category 2: patient education required; Triage category 3: intervention required; Triage category 4: critical need for intervention.

*Statistically significant difference, *p* < .05.

All of the correlations between PG-SGA score and PDQ-39 total and sub-scores were significant except for the stigma sub-score (Table 1). The strongest correlations were between PG-SGA score, total PDQ-39 score (*r*_*s*_ = 0.445, *p = *0.000), mobility sub-score (*r*_*s*_ *=* 0.520, *p* = 0.000), and activities of daily living sub-score (*r*_*s*_ = 0.412, *p* = 0.000).

### Phase II

**Figure 1:**
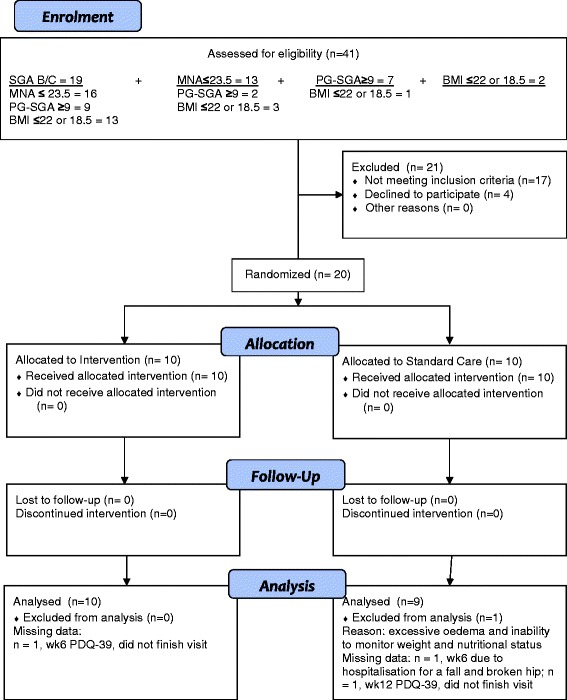
**CONSORT flow diagram of participant progression through the RCT of nutrition intervention in people with Parkinson’s disease.**

Twenty participants were eligible and agreed to participate in Phase II (Figure 1). One participant was excluded after data collection due to excessive oedema that resulted in an inability to monitor dry weight and weight changes (SC, n = 9; INT, n = 10). Data may have also been affected by the following: in the 2 weeks prior to the wk 12 visit, 1 INT injured her knee leaving her unable to mobilize well, 1 INT underwent esophageal surgery, and 1 INT suffered from a gastrointestinal illness.

Median age of the group was 69.0 (35–84) years with a median self-reported length of disease of 7.0 (1.5–26.5) years. The UPDRS III score in the SC group at BL was 18 (14–31) and in the INT group was 16.5 (9–30), and the change in the UPDRS III score in the SC group was 0 (-2–9) and 0.5 (-7–3) in the INT group; neither the BL value or change was significantly different between the groups. The LEDD (mg/kg) was 11.0 (0–23.3) mg/kg in the BL group and 8.4 (1.6–14.3) mg/kg in the INT group; this was also not significantly different. Over the 12-week period, the LEDD (mg/kg) decreased by 0.13 (-2.94–1.7) mg/kg in the SC group and increased by 0.50 (-0.3–6.39) mg/kg in the INT group, which represented a significant difference in the change between the groups (*U* = 70.00, *z* = 2.04, *p* = 0.43).

The groups were not significantly different at baseline for PDQ-39 scores, including the sub-scores. As a total group, the median PDQ-39 score decreased from 26 (9–61) to 23.5 (5–69), which is a clinically, but not statistically, significant improvement (*U* = 3.11, *p* = 0.211). The only sub-score to improve significantly for the total group was the EWB sub-score (*X*^*2*^(2) = 8.84, *p* = 0.012), with this change occurring between BL and wk 12 (*z* = 2.43, *p* = 0.045) (Table 3).

**Table 3:** **Baseline PDQ-39 score and sub-scores for each group and the median change in scores over the 12 weeks**

	**INT (n = 10)**	**INT median change over 12 wks**	**SC (n = 9)**	**SC median change over 12 wks**
PDQ-39 total score	22.5 (9 - 61)	-4 (-17 - 8)	28 (11 - 51)	-1.5 (-8 - 23)
Mobility	45 (5 - 78)	-2.5 (-28 - 30)	43.5 (2 - 88)	1.5 (-25 - 36)
Activities of daily living	25 (17 - 62)	-5 (-33 - 33)	42 (21 - 71)	0 (-13 - 17)
Emotional wellbeing*	8 (0 - 71)	-4 (-25 - 4)	29 (4 - 67)	-6.5 (-17 - 33)
Stigma	12.5 (0 - 56)	0 (-19 - 18)	3 (0 - 62)	0 (-6 - 12)
Social support	4 (0 - 75)	-4 (-13 - 8)	0 (0 - 50)	0 (-17 - 67)
Cognition	19 (0 - 38)	3 (-12 - 13)	34.5 (19 - 50)	0 (-19 - 18)
Communication	29 (8 - 75)	-4 (-25 - 9)	17 (0 - 67)	0 (-17 - 16)
Bodily discomfort	37.5 (0 - 67)	-4 (-25 - 9)	33 (0 - 92)	0 (-25 - 9)

*Abbreviations:* *PDQ-39* Parkinson’s disease questionnaire-39, *INT* Intervention group, *SC* Standard Care group.

*The score for the entire group improved significantly over the 12 week period, *p* < .05.

The median change in total PDQ-39 score over the 12 weeks was not significantly different between the groups (*U* = 33.00, *z* = -.894, *p = *0.356) nor was there a statistically significant change in either group over the intervention period (SC, *U* = .74, *p* = 0.690; INT, *U* = 4.38, *p* = 0.112). The change of 4 points in the PDQ-39 score in the INT group was clinically significant, while the improvement of 1.5 points in the SC group was not (Table 3).

When all PDQ-39 measurements for all participants across the 12 weeks were treated as one data set, there was a significant positive correlation between PG-SGA score and PDQ-39 score (*r*_*s*_ = .426, *p* = 0.002), indicating that poorer nutritional status was associated with poorer quality of life. In the linear mixed models analysis, neither the intervention group nor the intervention time period had a significant effect on PDQ-39 score (Table 4). Nor did gender or duration of disease at baseline demonstrate a significant effect on PDQ-39 scores. The log-transformed PG-SGA score bordered on statistical significance with poorer nutritional status (higher PG-SGA score) resulting in lower quality of life (higher PDQ-39 score), *p* = 0.051. Dichotomised H&Y score had a significant effect on PDQ-39 score with less severe disease resulting in better quality of life (lower PDQ-39 score), *p* = 0.014.

**Table 4:** **Predictors of quality of life (PDQ-39 score) over the 12-week intervention period**

	**Estimate**	**Standard error**	**Estimated marginal means**	***p*** **value**
Intercept	48.02	14.01	n/a	.003
Gender (male)	-0.70	7.27	37.94 (male)	.316
			38.64 (female)	
PD duration (years)	-0.80	0.69	n/a	.265
H&Y category (less severe)	-30.42	10.88	23.08 (less severe)	.014
			53.50 (severe)	
ln PG-SGA	5.37	2.67	n/a	.051
Group (SC)	7.51	7.27	40.38 (SC)	.316
			36.20 (INT)	
Time (BL)	4.33	2.38	39.47 (BL)	.079
			37.85 (wk 6)	
Time (wk 6)	2.87	2.50		.261
			37.56 (wk 12)	

*Abbreviations:* *BL* baseline, *H&Y* Hoehn & Yahr, *PD* Parkinson’s disease, *PDQ-39* Parkinson’s disease questionnaire-39, *PG-SGA* Patient-Generated Subjective Global Assessment, *SC* Standard Care.

AIC = 340.43; Estimated Marginal Means evaluated at ln PG-SGA 1.46 and PD duration 8.11 years.

## Discussion

The current paper aimed to explore the relationship between nutritional status and quality of life and also to investigate the effect of a nutrition intervention on quality of life in PD. In the current study, those who were malnourished reported lower quality of life across the majority of PDQ-39 domains, and there were significant relationships between nutritional status (PG-SGA scores) and PDQ-39 scores, indicating poorer quality of life in those with poorer nutritional status and supporting previous findings in a sample of PD patients attending a movement disorders clinic in Iran [26].

The strongest relationships existed between nutritional status and the areas of mobility and activities of daily living, and these were the domains that differentiated the well-nourished and malnourished. Similar results were recently reported in the study based in Iran, with the strongest correlation between the PDQ-39 mobility sub-score and nutritional status [26]. Therefore, perceived limitations in mobility may have the greatest relationship with nutritional status in PD. However, the precedence of these relationships cannot be determined owing to the cross-sectional nature of the present study. Poor mobility and the inability to carry out ADL can contribute to poor nutritional status [37]. On the other hand, malnutrition reduces mobility [38] and functional status, [18,39] and the ability to carry out activities of daily living plays an important part in quality of life [27,40]. Similarly, the finding of poorer social support in those with poorer nutritional status may be due to the fact that people with PD who live alone are more likely to be malnourished [30]. Social support plays an important role in maintaining nutritional status, particularly in a disease that results in considerable disability [37] and nutrition impact symptoms that can decrease food intake [25]. Therefore, these relationships should be explored further to determine the influence of the variables on each other.

There was also a positive relationship between changes in PG-SGA and PDQ-39 scores during the 12-week intervention period, regardless of the group (SC or INT) (Table 4). Although these results only bordered on significance, potentially because of the small sample size, the findings might indicate that improvements in nutritional status result in improvement in quality of life in PD.

Quality of life scores improved in both groups over the 12-week period, although these changes were not statistically significant. However, the INT group demonstrated a trend towards greater improvement than the SC group in the majority of domains, which resulted in clinically significant changes. The collaborative and personalised advice and the regular phone contact with a health professional in the INT group may have improved the group’s overall sense of well-being. It has been shown that patient involvement in medical treatment decisions increases the satisfaction with that treatment in people with PD, and this may also extend to nutrition-related interventions [41]. Participation in the study also resulted in significantly improved emotional well-being for the entire group. Perhaps even semi-regular contact with a health professional may improve the sense of being looked after.

Several factors may have resulted in a smaller effect of the intervention than may have otherwise been found. One is the small sample size, which is likely to have been underpowered to detect a difference. In Phase II particularly, this limits the ability to generalize the findings. However, given the limited evidence in this area, especially for nutrition interventions, this information helps to highlight the importance of nutritional status in the well-being of people with PD. Owing to the small sample size, more robust analysis could not be conducted to control for other confounding factors for either nutritional status or quality of life, such as motor function, depression, or other non-motor symptoms. Therefore, other factors such as depression, which is common with poor nutritional status, could have affected quality of life more than nutritional status *per se*.

There was also a large degree of variability in the responses as is demonstrated by the wide ranges within each PDQ-39 score. Within the INT group, there were three individuals who experienced changes in their health, unrelated to PD, in the weeks prior to the final data collection, resulting in poorer quality of life scores for them at that final time point. In addition, there were participants in both groups with missing data, particularly for the PDQ-39, because they did not want to finish the visit. This may have affected the comparison between the groups at those times. Furthermore, the quality of life in this sample was better than that reported in other studies, [14,42] which could limit the ability to affect a significant change in quality of life. The short study period of only 12 weeks may not have allowed for meaningful changes in quality of life to occur.

The choice of tool with which to measure quality of life may have also affected the results. It has been argued that the PDQ-39 measures health status (HS) rather than quality of life, where HS refers to function levels, and quality of life reflects internal experiences or the subjective evaluation of health [43]. The PDQ-39 is weighted toward physical symptoms and therefore may not appropriately measure health-related quality of life [44]. This may explain why the only significant improvement was found in the emotional well-being domain over the intervention period. Additionally, other important health related areas may be missing from the PDQ-39, such as those relating to motor fluctuations and medications [45] which may be influenced by body weight and nutritional status [46-48]. Finally, the use of self-reported PD diagnosis to identify participants may influence the generalisability of the results to patients with idiopathic PD, as not all patients are aware of their specific diagnosis (e.g., idjiopathic, drug-induced, progressive supranuclear palsy, multiple system atrophy).

## Conclusions

Malnourished people with PD presented with poorer quality of life than well-nourished participants. This was particularly evident in the areas of mobility and activities of daily living, both of which have been associated with poor nutritional status in other groups. Given the borderline significant relationship between improvements in nutritional status during the nutrition intervention and improvements in quality of life, it is likely that future studies conducted in larger samples would support this finding. Furthermore, the nutrition intervention resulted in improvements in emotional well-being. Attention to nutritional status is an important component of quality of life and therefore the total care of people with PD.
